# Strain induced topological phase transitions in monolayer honeycomb structures of group-V binary compounds

**DOI:** 10.1038/srep17980

**Published:** 2015-12-10

**Authors:** Yaozhuang Nie, Mavlanjan Rahman, Daowei Wang, Can Wang, Guanghua Guo

**Affiliations:** 1 School of Physics and Electronics, Central South University, Changsha, 410083 China

## Abstract

We present first-principles calculations of electronic structures of a class of two-dimensional (2D) honeycomb structures of group-V binary compounds. Our results show these new 2D materials are stable semiconductors with direct or indirect band gaps. The band gap can be tuned by applying lattice strain. During their stretchable regime, they all exhibit metal-indirect gap semiconductor-direct gap semiconductor-topological insulator (TI) transitions with increasing strain from negative (compressive) to positive (tensile) values. The topological phase transition results from the band inversion at the Γ point which is due to the evolution of bonding and anti-bonding states under lattice strain.

Two-dimensional (2D) topological insulators (TIs), also known as quantum spin Hall (QSH) insulators, have attracted much attention recent years due to their rich physics and promising applications in spintronics and quantum computations^1^,^2^,^3^,^4^. The QSH effect, which was first proposed in graphene^5^, describes the existence of the edge states on the sides of a 2D TI system. These edge states are supposed to consist of two counter-propagating oppositely spin polarized edge channels in the band gap of the material. However, the spin-orbit coupling (SOC) in graphene is too weak to open a gap large enough to support accessible QSH effect experimentally. Unlike graphene, its elemental analogues - silicene and germanene, show energy gaps because they have larger SOC due to their buckled honeycomb structures^6^. Therefore, researchers predict that QSH effect can be observed in an experimentally accessible temperature regime in both systems^7^. Other monolayer honeycomb structures of group-IV elements as well as III-V binary compounds have been systematically investigated based on first-principles calculations^8^. Theoretical studies show that many of these materials are 2D TIs at their equilibrium structures, including stanene^9^, InBi, GaBi, and TlBi^10^,^11^. Most recently, there has been rising interest in layered compounds of group-V elements^12^,^13^,^14^,^15^,^16^. Elemental phosphorus occurs in at least 10 allotropic forms. Among them, blue phosphorous has the same layered structure as bulk arsenic, antimony, and bismuth. Their corresponding monolayer materials have the same buckled honeycomb structure as silicene and germanene^12^,^17^. Monolayer bismuth is reported a 2D TI, while other monolayer buckled honeycomb structures of group-V elements are conventional semiconductors^18^,^19^,^20^. Furthermore, recent studies indicate that band topology in these 2D materials could be altered by chemical adsorptions^21^,^22^,^23^, external electric field^24^,^25^, and lattice strain^25^,^26^,^27^. Under lattice strain, monolayer honeycomb structure of P, As, and Sb all exhibit trivial semiconductor to TI transition^27^. The band inversion at the Γ point is identified as the driving force for the topology change. However, what makes the band inversion at the Γ point under lattice strain is still unclear. We also note that there is few report of monolayer honeycomb structures of group-V binary compounds, although bulk AsSb has the same layered structure as bulk arsenic, antimony, and bismuth^28^.

In this paper, we study monolayer honeycomb structures of group-V binary compounds (except nitrogen) by first-principles calculations. We investigate the stability of these new 2D materials by studying phonon dispersion and molecular dynamical simulations, and calculate their band structures under lattice strains. We find all of them are stable semiconductors with indirect or direct energy gaps. Under strain, they all exhibit metal-indirect gap semiconductor-direct gap semiconductor-topological insulator (TI) transitions. It is noteworthy that the mechanism of topological transition is due to the evolution of bonding and anti-bonding states under lattice strain.

## Results

**Figure 1:**
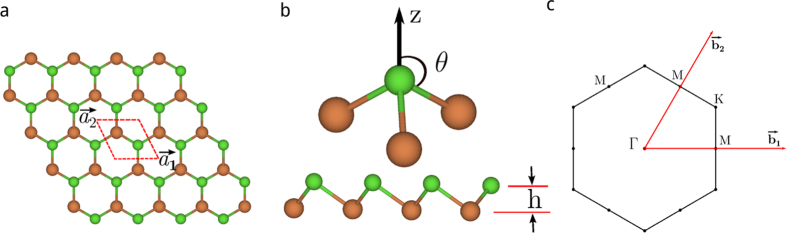
The lattice geometry of buckled honeycomb structure. (**a**) Top view of buckled honeycomb structure. Note that A sublattice and B sublattice (denoted by green and brown color, respectively) are not coplanar. 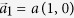, 
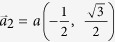
. (**b**) Side view of buckled honeycomb structure. Definition of the angle *θ* and buckling parameter h. (**c**) Brillouin zone and specific symmetry points. 
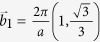
, 
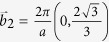
.

Bulk AsSb, or arsenic antimonide, has the same layered crystal structure as arsenic, antimony, and bismuth (space group 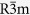, No. 166), with the intermediate values of lattice parameters of arsenic and antimony, *a* = 4.025 Å, *c* = 10.84 Å. Like monolayer As and Sb, we consider monolayer AsSb consisting of a layer of As and a layer of Sb. This new material has the same structure as silicene and germinene, i.e. the buckled honeycomb structure, as shown in Fig. 1. We perform geometry optimization for such a monolayer AsSb, obtain the lattice constants *a* = 3.867 Å, and calculate the binding energy. Motivated by this, we consider all possible group-V binary compounds (except nitrogen) with the same structure. The optimized lattice constants, buckled parameters, and binding energies are calculated, and the corresponding parameters of monolayer P, As, Sb, and Bi are also given for the sake of comparison (Table S1).

**Figure 2:**
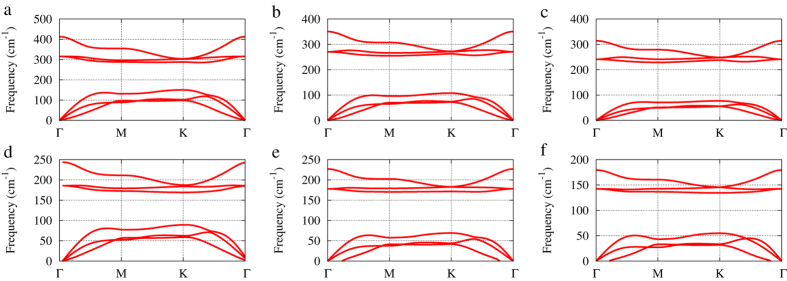
Phonon dispersion curves of buckled honeycomb structures of group-V binary compounds. (**a**) PAs. (**b**) PSb. (**c**) PBi. (**d**) AsSb. (**e**) AsBi. (**f**) SbBi.

To check the stability, we calculate the phonon dispersion of these monolayer binary compounds. Absence of negative frequencies of the calculated phonon dispersion indicates the stability of monolayer PAs, PSb, PBi, and AsSb (see Fig. 2). Note that ZA branch (out of plane acoustical modes) becomes soft and get imaginary frequencies near the Γ point for AsBi and SbBi. This situation has been interpreted as the instability against long-wavelength transversal waves. It is believed such instability can be removed by defects, such as ripples, or finite size sheets, which do not allow these waves^6^,^8^. On the other hand, the ZA imaginary frequencies around Γ point also depends on the mesh size used in the calculations. It may be an artifact of the mesh size since the interatomic forces related with ZA modes decay rapidly^6^,^8^. The MD simulations also show the stability of these 2D compounds (including AsBi and SbBi) because no structural collapse happens during the simulations (Supplementary Information 1).

**Figure 3:**
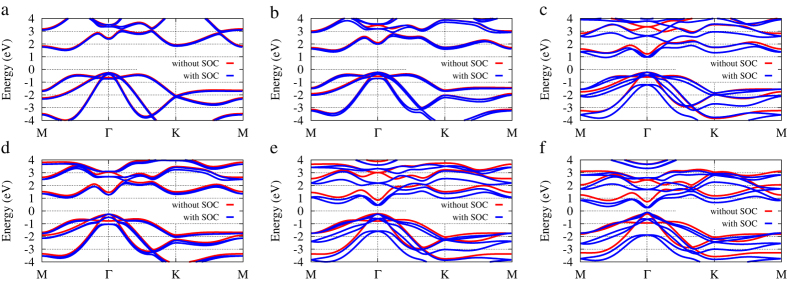
Energy bands of binary compounds with buckled honeycomb structure. (**a**) PAs. (**b**) PSb. (**c**) PBi. (**d**) AsSb. (**e**) AsBi. (**f**) SbBi.

Figure 3 shows band structures of these binary compounds. The band structures of monolayer phosphorous, arsenic, antimony, and bismuth are also given for comparison (Figure S1). The calculated band structures (without SOC) show similarities between different systems. This is not surprising, since these elements are chemically similar. Taking SOC into account, the number of bands of binary compounds is doubled due to the nonsymmetric structure and SOC. Note that SOC just modifies the band gaps but does not change band orders for these binary compounds, P, As, and Sb. However, the band structure of Bi differs from others when SOC is included. The parity of the states that form the valence and conduction bands is reversed at the Γ point. This exchange of bands is the consequence of the increased SOC in Bi, and Bi is reported a 2D topological insulator^18^. On the contrary, other 2D group-V elemental materials and binary compounds with the same buckled honeycomb structure are conventional insulators. Take monolayer AsSb for example, the calculated band structure (without SOC) shows it is a semiconductor with a indirect gap of 1.48 eV, while a gap of 1.73 eV at Γ. The valence band maximum (VBM) is at the Γ point, and the conduction band minimum (CBM) is at *k* = (0.132, 0) point. The band structures (without SOC) of binary compounds containing Bi possess direct gaps, while others show indirect gaps.

**Figure 4:**
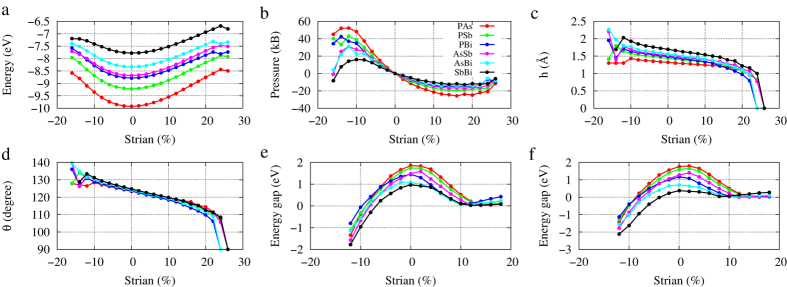
Some parameters as functions of lattice strain. (**a**) Total energy. (**b**) In-plane pressure. (**c**) Buckling parameter h. (**d**) Angle *θ*. (**e**) Energy gap (without SOC). (**f**) Energy gap (with SOC).

We perform strain engineering to these 2D binary compounds. Figure 4 shows calculated energies, pressures, buckling parameters, and energy gaps as functions of the strain. From the place where the stress maximum (minimum) occur we can determine the stretchability of the material. Take AsSb for example, the stretchability is about −12% for press, and 18% for stretch. During these domain, buckling parameters h and *θ* change almost linearly. When strain is larger than 26%, h drops to zero and *θ* equals 90°, and corresponding energy also changes rapidly. It means the system becomes planar honeycomb structure as graphene. Band gaps shown in Fig. 4e (without SOC) and Fig. 4f (with SOC) indicate that the system is metallic when strain is between −12% and −8%; when strain is between −8% and 2%, the system is a indirect insulator; the system becomes a direct semiconductor with a gap at the Γ when strain is larger than 2%. The band gap decreases with increasing strain and closes at about 12% strain, then reopen when strain is larger than 12%. Band structures of other binary compounds as well as P, As, and Sb under strain have similar characteristics (Figure S2–S7).

**Figure 5:**
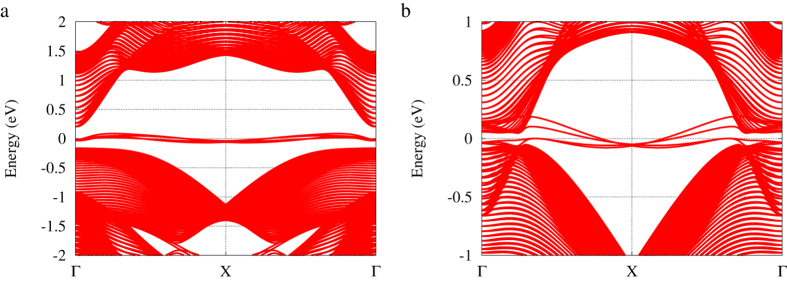
Band structures of AsSb nanoribbon under different lattice strains. (**a**) Band structure at 8% lattice strain. (**b**) Band structure at 16% lattice strain.

The existence of topological protected gapless edge states is one of the most important consequences of 2D topological insulators. To verify the edge states, we construct zigzag ribbons with 88 atoms in a unit cell (about 156.72 Å and 168.33 Å wide), under strain 8% and 16%, respectively. Figure 5 shows the calculated band structures. We observe both ribbons show band gaps, and inside the gap, as expected, we observe four states, corresponding to two spin-splitting edge states from either side of the ribbon. AsSb ribbon has topologically protected gapless edge states under 16%. Although gapless edge states are also shown under 8%, they are not topologically protected.

## Discussion

To get a better understanding of the mechanism underlying the band inversion, we investigate the band structure evolution at the Γ point. Take AsSb for example, As and Sb have five electrons in the outer shell. For each atom, two electrons occupy s orbital and three electrons occupy p orbitals. They form five energy bands below the Fermi energy. The two s bands are much lower than the three p bands. The three p bands of two atoms in the cell form six bands with three below the Fermi energy and three above the Fermi energy. According to our first-principles calculations, ignoring normalization factors, these six bands around the Γ point are mainly denoted by 
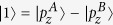
, 
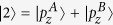
 (in fact, there are some 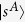 and 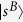 components in 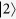), 
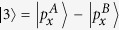
, 
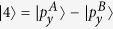
, 
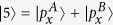
, and 
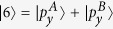
. A denotes As atom and B Sb atom. The 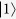, 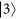, and 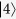 are below the Fermi energy while 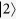, 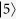, and 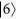 are above the Fermi energy. When SOC is not included, 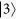 and 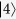 are degenerated at the Γ point, and so are 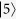 and 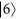.

**Figure 6:**
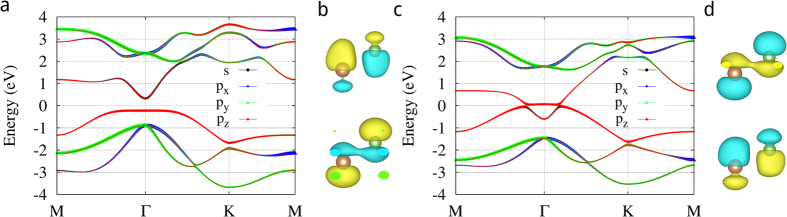
Band structures of AsSb at different lattice strains, HOMO and LUMO orbitals at the Γ point. (**a**) Band structure at 8% lattice strain. (**b**) HOMO 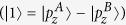 and LUMO 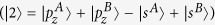 orbitals at the Γ point at 8% lattice strain. Green and brown spheres represent As atom and Sb atom, respectively. Yellow (green) color in orbitals means positive (negative) phase. (**c**) Band structure at 16% lattice strain. (**d**) HOMO 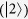 and LUMO 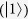 orbitals at the Γ point at 16% lattice strain.

Under ambient conditions, 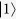 is below 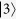 and 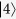. When strain reaches up to 2%, the indirect gap becomes a direct gap. When strain is 3%, 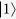, 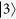, and 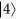 have the same energy. When strain is beyond 3%, the two bands below and above the Fermi energy at the Γ point is constructed by 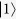 and 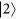, respectively. Figure 6 shows the band structures of monolayer AsSb at strain of 8% and 16% respectively, and the band composition. We see the band inversion at the Γ according to Fig. 6a,c. Because h is relatively large when strain is small, the low half of 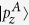 is close to the upper half of 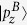, their coupling is bonding when they are out of phase, while their coupling is anti-bonding when they are in phase. Therefore 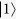 has a lower energy than 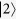. Figure 6b shows 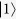 and 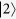, corresponding to VBM and CBM, respectively. In other words, 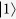 is the highest occupied molecular orbital (HOMO) at the Γ point, while 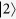 is the lowest unoccupied molecular orbital (LUMO) at the Γ point. Note that 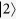 shows 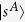 and 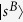 components. With further increase of strain, h decreases and the low half of 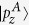 is move from upper half of 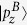 to low half of 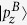, then energy of 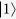 increases and 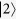 decrease. When strain is about 12%, 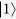 and 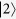 have the same energy and the gap closes. When strain is larger than 12%, the low half of 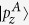 is closer to the low half of 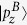 because of small h, then their coupling is bonding when they are in phase, while their coupling is anti-bonding when they are out of phase, therefore 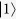 has a higher energy than 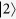. Now 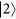 is HOMO at the Γ point, while 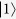 is LUMO at the Γ point, and the band inverts, as shown in Fig. 6c,d.

When A and B are the same element such as P, As, and Sb monolayer, the system has inversion symmetry. 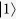 and 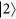 orbitals have even and odd parity, respectively. The 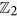 invariant can be determined from the parities of the occupied states at the time-reversal invariant momenta in the Brillouin zone^29^. There are four time-reversal invariant momenta for buckled honeycomb structure, i.e., one Γ and three M points, as shown in Fig. 1c. The band inversion at the Γ means that topological invariants 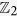 changes. Directly parity counting at time reversal invariant momenta shows that the 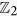 changes from 0 to 1 for monolayer P, As, and Sb, when stain is larger than 12% (Table S2). We note that As and Sb are reported to exhibit topological phase transition at tensile strain 6%^27^. However, these values are estimated according to bulk As and Sb lattice constants *a*, which is about 6% longer than those of corresponding 2D lattice constants, respectively^26^. In other words, our results are in agreement with theirs.

When A and B are different elements, the inversion symmetry is broken. Then 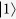, 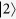, and other states, are not the eigenstates of parity. Then it is not possible to determine the 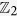 invariant from the symmetry of the occupied states at time-reversal invariant momenta. To check the topological invariants, we adopt the method proposed by Soluyanov and Vanderbilt^30^. The method is to track the largest gap between Wannier charge centers (WCCs). The path following the largest gap between WCCs cross the WCCs bands a number of times that is equal, mod 2, to the 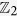 invariant. The calculated 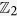 indices show AsSb is a conventional insulator under 8% strain and a topological insulator under 16% (Figure S8).

The mechanism of the band inversion at the Γ point is the different response of energies of 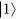 and 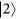 to lattice strain, which is due to the evolution of bonding and anti-bonding states under lattice strain. The origin of the nontrivial topology in buckled honeycomb structures of group-V binary compounds (as well as monolayer P, As, and Sb with the same structure) results from the band inversion due to lattice strain. It is neither like the band inversion at the 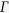 point in monolayer Bi, HgTe quantum well, and *Bi*_2_*Se*_3_, which is due to SOC, nor like that in silicene and germanene, which results from massive Dirac cone and there is no band inversion. In the case of these 2D honeycomb structures of group-V binary compounds, SOC just modifies energy gaps, as shown in Fig. 4e,f. For example, monolayer AsSb, PBi, and SbBi exhibit topological energy gaps of 77, 333, and 69 meV under 16% lattice strain without including SOC, respectively. Taking SOC into account, the corresponding energy gaps are 50, 29, and 247 meV, respectively. However, topological protected gapless edge states do not exist unless SOC is taken into account.

Note that the existence of 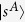 and 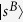 components in 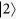 does not change the mechanism of the band inversion mentioned above. However, it makes the difference between band dispersions of 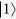 and 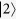 near the Γ point. A remarkable feature is that the 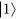 band becomes flatter with increasing strain, in particular when it gets close to the Fermi energy. In fact, the 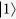 band around the Γ point can be approximately given by (Supplementary Information 2)


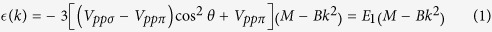


When the energy of 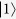 equals to that of 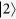, we have *E*_1_ = 0. It means the valence band becomes flat. On the contrary, the existence of 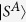 and 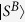 components in 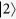 makes its band dispersive. In recent years, lattice models with flat bands have attracted attention for a number of reasons, among them are enhanced interaction effects^31^.

In conclusion, we predict that monolayer honeycomb structures of group-V binary compounds are stable according to phonon dispersion and molecular dynamical simulations. These new 2D materials are semiconductors under ambient conditions. Monolayer PBi, AsBi, and SbBi possess direct gaps, while PAs, PSB, and AsSb possess indirect gaps. Under lattice strain, they show topological phase transitions according to calculated 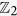 invariants. By constructing ribbons, we show topologically protected edge states exist at nontrivial topology phase. Analysis of the band structure evolution at the Γ point indicates that the band inversion is due to the evolution of bonding and anti-bonding states under lattice strain. This mechanism of topological transition under lattice strain is different from that of previously studied 2D TIs. We hope that our work will promote the research aiming at the synthesis of these new 2D materials and the search for new 2D TIs.

## Methods

Our first-principles calculations are carried out in the framework of density-functional theory (DFT)^32^ within the Perdew-Burke-Ernzerhof generalized gradient approximation (GGA)^33^ implemented in the ABINIT codes^34^. Norm-conserving HGH pseudopotentials^35^ and the plane-wave cutoff energy of 30 Hartree are used. For monolayer binary compounds, a supercell with a vacuum space of 20 Å along the z-direction is employed with a 12 × 12 × 1 k-point mesh. Both the lattice parameters and the positions of all atoms are relaxed until the force is less than 1 meV/Å. The phonon frequencies are computed using density-functional perturbation theory (DFPT)^36^ with a 6 × 6 × 1 q-point mesh. We perform canonical molecular dynamics (MD) simulation at 300 K with a supercell of 72 atoms. The length of time-step of 3 fs and simulations with 500 steps are executed.

## Additional Information

**How to cite this article**: Nie, Y. *et al*. Strain induced topological phase transitions in monolayer honeycomb structures of group-V binary compounds. *Sci. Rep.* **5**, 17980; doi: 10.1038/srep17980 (2015).

## Supplementary Material

Supplementary Information

Supplementary Video 1

Supplementary Video 2

Supplementary Video 3

Supplementary Video 4

Supplementary Video 5

Supplementary Video 6
