# Supplementary material for: Strain induced topological phase transitions in monolayer honeycomb structures of group-V binary compounds
Source: Sci Rep. 2015 Dec 10;5:17980. doi: 10.1038/srep17980 (PMC4674708; doi:10.1038/srep17980)
Supplement: Supplementary Information [file srep17980-s1.pdf]

Supplementary Information for:
Strain induced topological phase transitions in monolayer honeycomb structures of group-V binary compounds

Yaozhuang Nie,* Mavlanjan Rahman, Daowei Wang, Can Wang, and Guanghua Guo†

School of Physics and Electronics, Central South University, Changsha, 410083 China

*Corresponding Author. yznie@csu.edu.cn

†Corresponding Author. guogh@mail.csu.edu.cn

I. MOLECULAR DYNAMICS SIMULATIONS

The following movies illustrate the dynamics of these 2D binary compounds at $T = 300K$.
PAs-300K-top.avi represents the infinite PAs monolayer at $T = 300K$ in top view;
PSb-300K-top.avi represents the infinite PSb monolayer at $T = 300K$ in top view;
PBi-300K-top.avi represents the infinite PBi monolayer at $T = 300K$ in top view;
AsSb-300K-top.avi represents the infinite AsSb monolayer at $T = 300K$ in top view;
AsBi-300K-top.avi represents the infinite AsBi monolayer at $T = 300K$ in top view;
SbBi-300K-top.avi represents the infinite SbBi monolayer at $T = 300K$ in top view.

II. ENERGY OF BANDS AT Γ POINT

The three p bands of two atoms in the cell form six bands with three below the Fermi energy and three above the Fermi energy at Γ point, which are denoted by $|1\rangle = |p_z^A\rangle - |p_z^B\rangle$, $|2\rangle = |p_z^A\rangle + |p_z^B\rangle$ (if ignoring $|s^A\rangle$ and $|s^B\rangle$ components in $|2\rangle$), $|3\rangle = |p_x^A\rangle - |p_x^B\rangle$, $|4\rangle = |p_y^A\rangle - |p_y^B\rangle$, $|5\rangle = |p_x^A\rangle + |p_x^B\rangle$, and $|6\rangle = |p_y^A\rangle + |p_y^B\rangle$. The energies of these states are given by Eq.1-4, respectively.

$$E_1 = -3[(V_{pp\sigma} - V_{pp\pi})\cos^2\theta + V_{pp\pi}] \tag{1}$$

$$E_2 = 3[(V_{pp\sigma} - V_{pp\pi})\cos^2\theta + V_{pp\pi}] \tag{2}$$

$$E_3 = E_4 = -\frac{3}{2}[(V_{pp\sigma} - V_{pp\pi})\sin^2\theta + 2V_{pp\pi}] \tag{3}$$

$$E_5 = E_6 = \frac{3}{2}[(V_{pp\sigma} - V_{pp\pi})\sin^2\theta + 2V_{pp\pi}] \tag{4}$$

At the critical point of topological phase transition, let the energy of $|1\rangle$ equals to that of $|2\rangle$, we have $E_1 = E_2 = 0$. The angle θ depends on matrix elements $V_{pp\sigma}$ and $V_{pp\pi}$.

When $|1\rangle$, $|2\rangle$ and $|3\rangle$ are degenerated, let $E_1 = E_3$, we get $\cos^2(\theta) = 1/3$, or $\theta \approx 125.3°$. The point is that it is independent of the matrix elements $V_{pp\sigma}$ and $V_{pp\pi}$. Our calculations based on LDA, GGA, and HSE06 all give the same θ, although $\theta \approx 122.7°$ for AsSb based on first-principles calculations. On the contrary, the critical point of topological phase transition is different for different calculation schemes such as LDA, GGA, and HSE06. Because different calculation schemes give different matrix elements $V_{pp\sigma}$ and $V_{pp\pi}$.

Table S1

The results of optimized geometries obtained by DFT with PBE. Lattice constants a, nearest-neighbour distance d, bulking hight h, bulking angle θ, and cohesive energy E_c are given.

Table S2

The total parity at Γ and three M points, and $\mathbb{Z}_2$ index of monolayer As under 8% strain and 16% strain. P and Sb have the same values as As. Note that the three M points are not all the same because the center of inversion defined by our unit cell is not at the center of a hexagon.

TABLE I:

	a(Å)	d(Å)	h(Å)	θ(deg)	E_c(eV/atom)
P	3.281	2.261	1.234	123.08	-3.479
As	3.605	2.508	1.399	123.90	-2.965
Sb	4.121	2.892	1.643	124.64	-2.623
Bi	4.331	3.045	1.739	124.81	-2.438
PAs	3.460	2.394	1.319	123.44	-3.177
PSb	3.733	2.590	1.435	123.66	-2.961
PBi	3.881	2.688	1.483	123.51	-2.785
AsSb	3.867	2.699	1.516	124.18	-2.794
AsBi	3.993	2.783	1.559	124.07	-2.663
SbBi	4.226	2.969	1.692	124.75	-2.520

TABLE II:

8% lattice strain						16% lattice strain					
Γ	+	-	+	+	+	Γ	+	-	+	+	-
M_1	+	-	-	+	+	M_1	+	-	-	+	+
M_2	+	-	-	+	+	M_2	+	-	-	+	+
M_3	-	+	+	-	-	M_3	-	+	+	-	-
$\mathbb{Z}_2$ invariant $\nu = 0$						$\mathbb{Z}_2$ invariant $\nu = 1$					

Figure S1

Band structures of buckled honeycomb structures of group-V elements.

Figure S2

Band structures of PAs at different lattice strains.

Figure S3

Band structures of PSb at different lattice strains.

Figure S4

Band structures of PBi at different lattice strains.

Figure S5

Band structures of AsSb at different lattice strains.

Figure S6

Band structures of AsBi at different lattice strains.

Figure S7

Band structures of SbBi at different lattice strains.

Figure S8

Evolution of AsSb WCCs (circles) at different lattice strains, red rhombus marks midpoint of the largest gap. The gap makes no jumps in Fig.8(a), while the center of the largest gap jumps over 1 WCCs in Fig.8(b). Thus the $\mathbb{Z}_2$ indices given by Fig.8(a) and Fig.8(b) are 0 and 1, respectively.

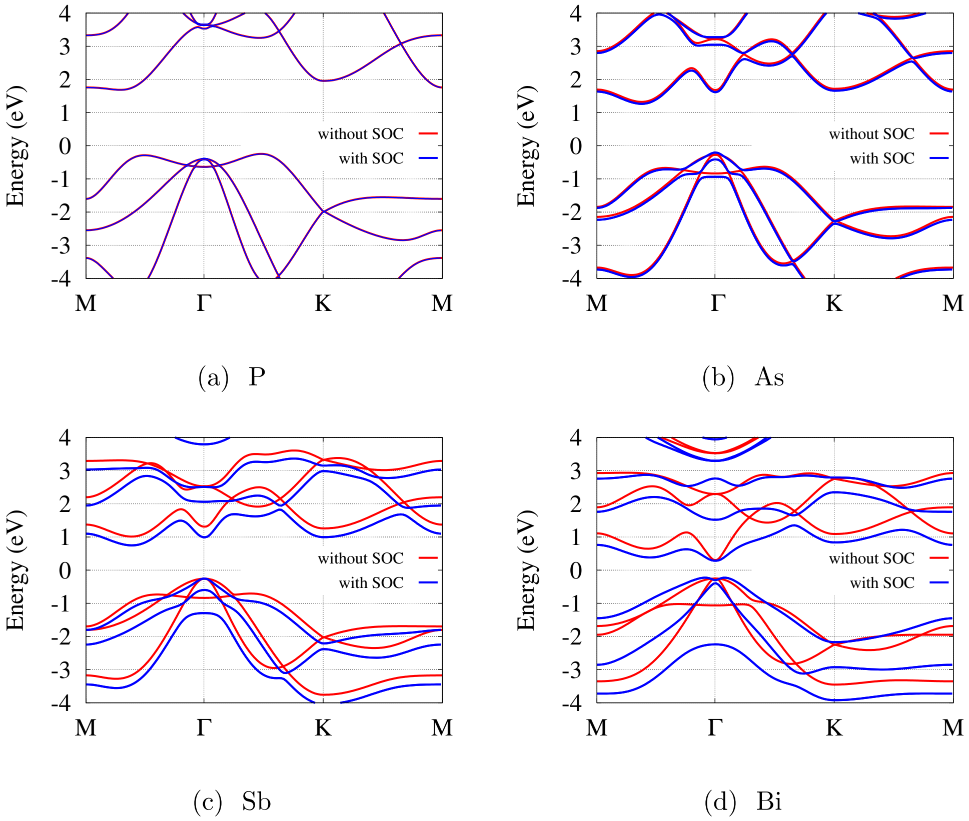

(a) P

(b) As

(c) Sb

(d) Bi

FIG. 1:

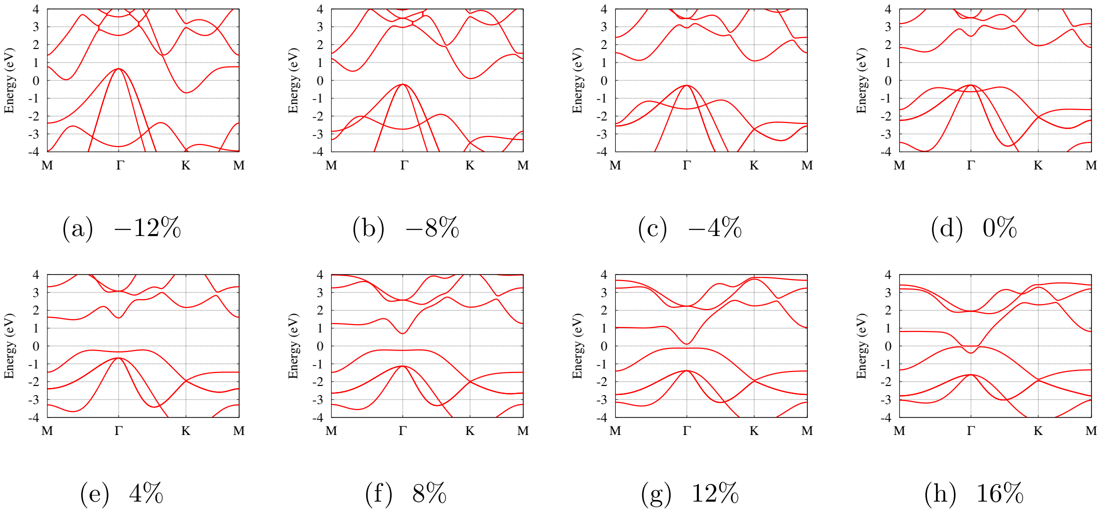

(a) −12% (b) −8% (c) −4% (d) 0%

(e) 4% (f) 8% (g) 12% (h) 16%

FIG. 2:

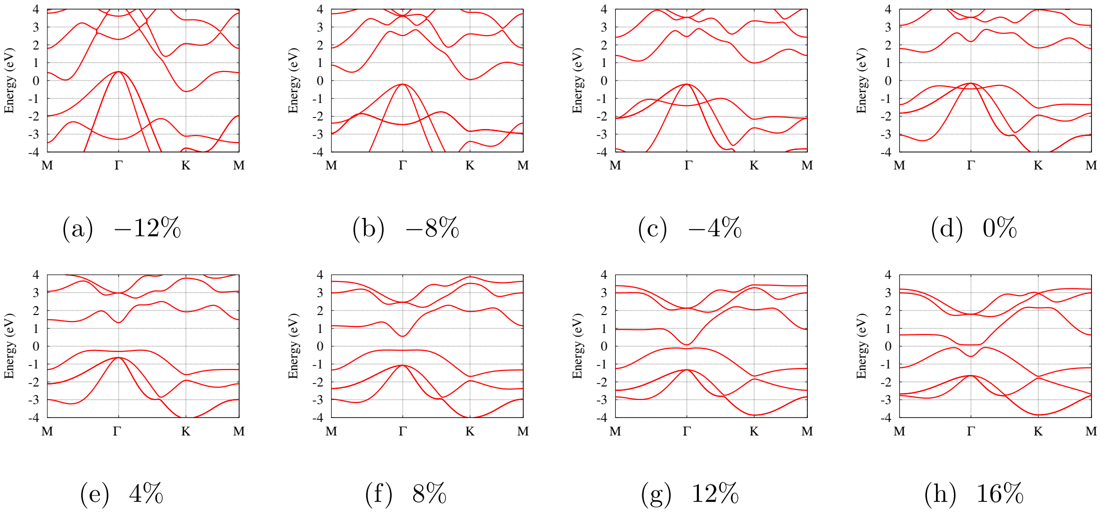

(a) −12% (b) −8% (c) −4% (d) 0%

(e) 4% (f) 8% (g) 12% (h) 16%

FIG. 3:

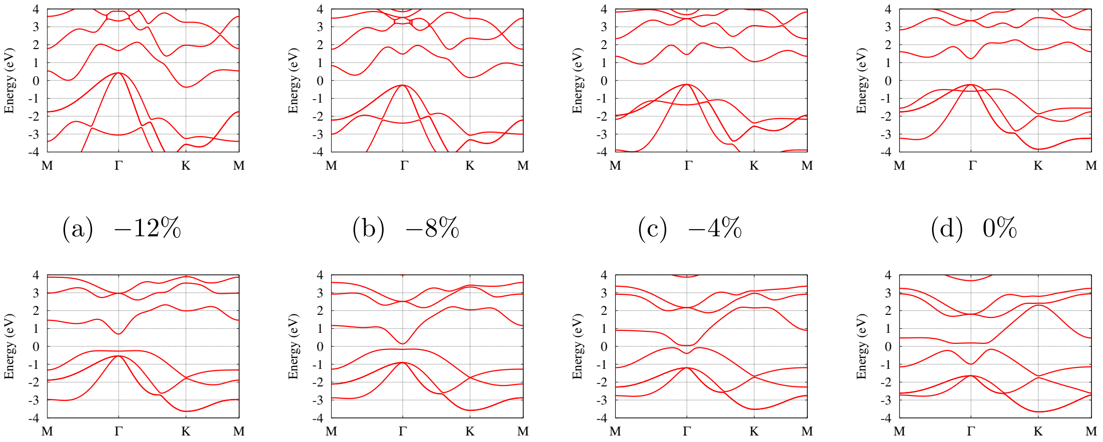

(a) −12% (b) −8% (c) −4% (d) 0%

(e) 4% (f) 8% (g) 12% (h) 16%

FIG. 4:

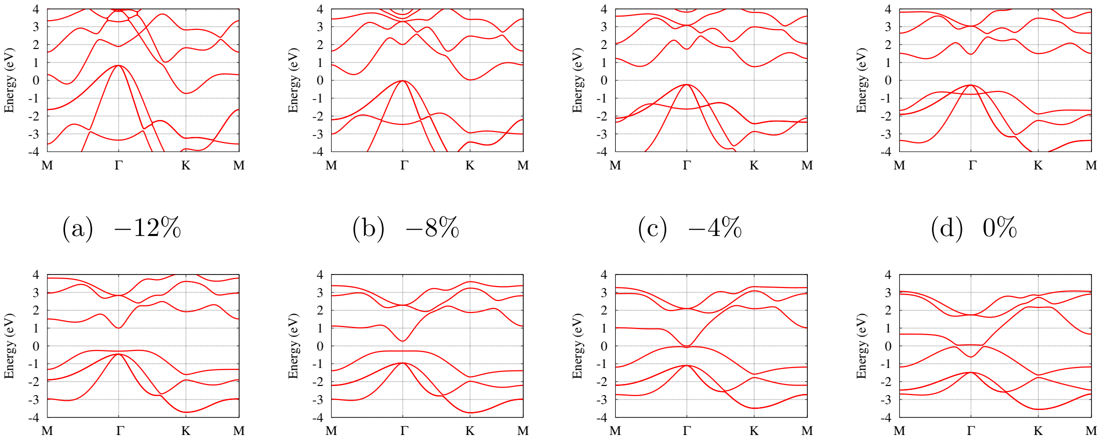

(a) −12% (b) −8% (c) −4% (d) 0%

(e) 4% (f) 8% (g) 12% (h) 16%

FIG. 5:

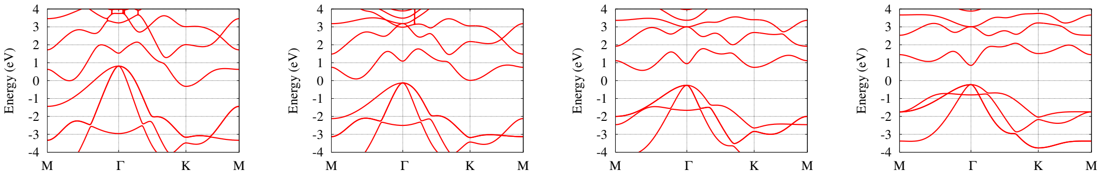

(a) −12% (b) −8% (c) −4% (d) 0%

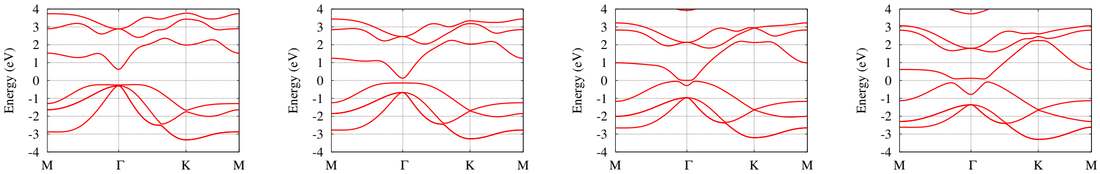

(e) 4% (f) 8% (g) 12% (h) 16%

FIG. 6:

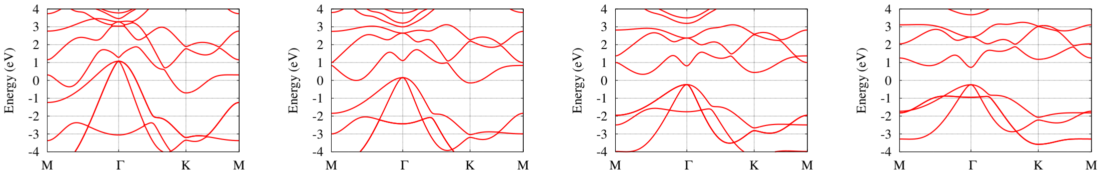

(a) −12% (b) −8% (c) −4% (d) 0%

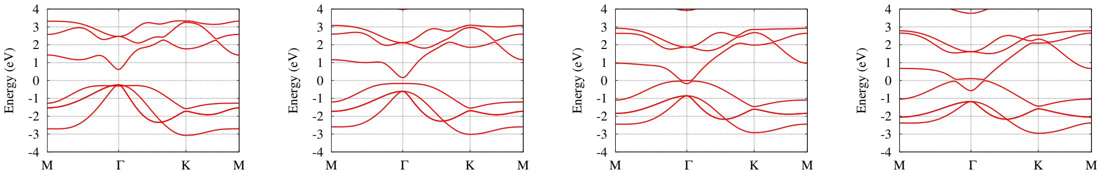

(e) 4% (f) 8% (g) 12% (h) 16%

FIG. 7:

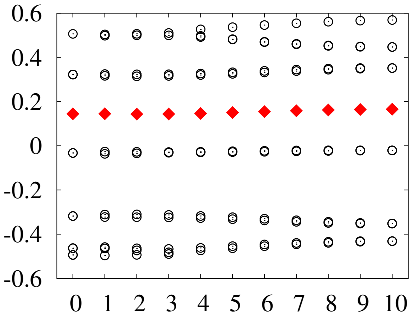

(a) 8%

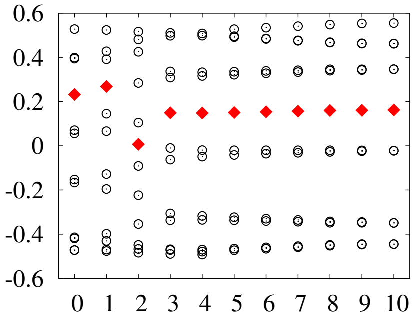

(b) 16%

FIG. 8:
